# Beware of Primate Life History Data: A Plea for Data Standards and a Repository

**DOI:** 10.1371/journal.pone.0067200

**Published:** 2013-06-24

**Authors:** Carola Borries, Adam D. Gordon, Andreas Koenig

**Affiliations:** 1 Department of Anthropology, Stony Brook University, SUNY, Stony Brook, New York, United States of America; 2 Department of Anthropology, University at Albany, SUNY, Albany, New York, United States of America; Texas A&M University, United States of America

## Abstract

Life history variables such as the age at first reproduction and the interval between consecutive births are measures of investment in growth and reproduction in a particular population or species. As such they allow for meaningful comparisons of the speed of growth and reproduction between species and between larger taxa. Especially in primates such life history research has far reaching implications and has led for instance to the “grandmother hypothesis”. Other links have been proposed with respect to dietary adaptations: Because protein is essential for growth and one of the primary sources of protein, leaves, occurs much less seasonally than fruits, it has been predicted that folivorous primates should grow faster compared to frugivorous ones. However, when comparing folivorous Asian colobines with frugivorous Asian macaques we recently documented a longer, instead of a shorter gestation length in folivores while age at first reproduction and interbirth interval did not differ. This supports earlier findings for Malagasy lemurs in which all life history variables tested were significantly longer in folivores compared to frugivores. Wondering why these trends were not apparent sooner, we tried to reconstruct our results for Asian primates with data from four popular life history compilations. However, this attempt failed; even the basic, allometric relationship with adult female body mass that is typical for life history variables could not be recovered. This negative result hints at severe problems with data quality. Here we show that data quality can be improved significantly by standardizing the variables and by controlling for factors such as nutritional conditions or infant mortality. Ideally, in the future, revised primate life history data should be collated in a central database accessible to everybody. In the long run such an initiative should be expanded to include all mammalian species.

## Primate Life History

Mammalian life history is composed of a few key variables characterizing the speed of growth (e.g., gestation length, age at weaning, age at first reproduction), the speed of reproduction (e.g., litter size, interval between consecutive births), and the duration of reproduction (i.e., life span minus age at first reproduction) [1]. Especially in the Order Primates the study of life history has been an active research field for several decades now with implications reaching beyond “mere” evolution of life histories: for example, it provides the foundation for the reconstruction of life history values for extinct taxa [2]–[4], it helps explain dental development [5], [6], the genetic basis for the timing of birth [7], the evolution of non-maternal care [8], and it has led to the grandmother hypothesis [9]. Primate life history research is also fundamental in identifying the peculiarities of our own species with its slow growth but fast reproductive rate and a very slow aging process [10], [11].

Given this general importance of life history data and with the scientific ideals of transparency and repeatability in mind, one would assume that researchers have long since agreed upon life history definitions and are using reliable and accurate data stored in standardized repositories. However, while various databases exist, standards for data quality and specification are lacking. In the following we will show some of the consequences of this lack and argue that data specificity, particularly in the form of additional population-specific information (which we collectively refer to here as metadata), are needed for future progress in life history research. Essentially we call for a standardized database of highest possible quality – a new life history repository similar to GenBank®. Because the problems disclosed for primate life history data are not unique to this taxonomic group, a repository for all mammals seems necessary.

## Existing Databases

Past life history analyses either relied on data collected by the authors or compiled from the primary literature [5], [12], but mostly previously published compilations were used. For primates, a database for mammals (Ernest [13]) is frequently used (recently by [14]–[16]) or PanTHERIA [17], an extensive compilation also for mammals provides the foundation for many studies (for example [18]–[23]). Furthermore, a life history compilation specifically for primates (Kappeler & Pereira [24]) has been used repeatedly [25]–[28]. Most recently, a new database went online that covers many aspects of nonhuman primate behavioral ecology (“All the World’s primates” Rowe & Myers [29]). To our knowledge its life history data have not yet been used in a published analysis, but this will be just a matter of time [30].

Generally, when using these compilations one proceeds from the assumption that the data are reliable and accurate (*sensu* Martin & Bateson [31]). However, some of the databases in use were compiled several decades ago [32], [33] and newer, more accurate data have become available in the meantime. The difference between more recent and older life history values for the same species can be very large, up to twofold for example for gestation length.

### Sources of Variation in Life History Measures

There are a number of factors that can affect the values of life history variables for a given population and which can potentially obscure underlying biological patterns. Here we draw attention to two in particular: differences in the operational definition of how a life history variable is measured, and population-specific covariates that impact on life history.

## Variation in Measurement Definitions

### Gestation length

**Figure 1:**
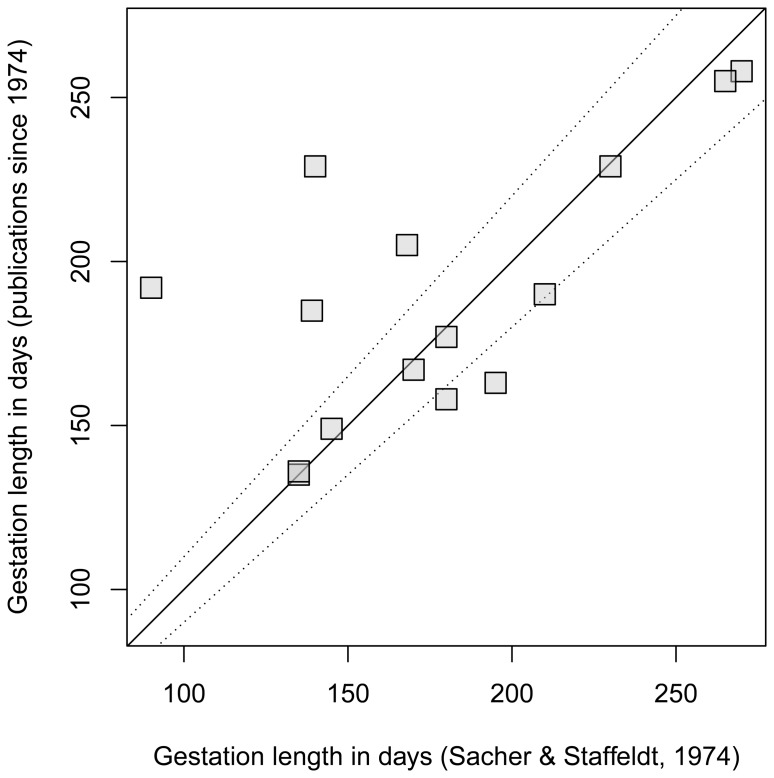
Gestation lengths for 15 nonhuman primates in 1974 and today. If past and present values were identical they would fall on the solid line (y = x). Points between the dotted lines indicate more recent values that are between 90% to 110% of the 1974 value. Data from Table 1.

Within a given species, gestation length is perhaps the least variable life history trait [34], [35] likely because its duration is shorter than most other traits, which reduces the magnitude of any kind of influence. Overall, it seems reasonable to assume gestation length to be a rather fixed value and, if measured accurately, new, more recent data for the same species should not differ by much. This is probably why fairly old compilations of gestation length are still in use today. For example, recent comparative studies [7], [36] used the values published in 1974 for 15 nonhuman primates by Sacher and Staffeldt [32]. However, when we compared this compilation with gestation lengths published more recently, only two of 15 were very similar while seven of 15 (47%) deviated by at least 10% (*Hylobates* spp.) and up to over 200% (*Nycticebus* spp.; Table 1, Fig. 1). Although the two datasets were significantly correlated (Pearson’s *r* = 0.639, *P*<0.02), the correlation coefficient was low considering that one of the leading methods textbooks suggests higher values: “… where measurement is straightforward, reliability should be well above 0.7″ (page 78 [31]).

**Table 1:** Gestation length [days] in nonhuman primates as provided in Sacher & Staffeldt [32] compared to more recent values, for which conceptions were determined based on hormonal analysis or other physiological measures.

Species^1^	Sacher & Staffeldt [32]	More recent value	[More recent value] as proportion of [Sacher & Staffeldt’s value]	Reference(s) for more recent value
*Lemur catta*	135	135	1.00	[93]
*Nycticebus coucang*	90	192	**2.13**	[94]
*Otolemur crassicaudatus*	135	136	1.01	[95]
*Alouatta paliatta*	139	185	**1.33**	[96], [97]
*Cebus capucinus*	180	158	***0.88***	[98]
*Ateles* spp.^2^	140	229	**1.64**	[99]
*Callithrix geoffroyi*	145	149	1.03	[100]
*Macaca mulatta* 3	170	167	0.98	[101]
*Papio* spp.^2^	180	177	0.98	[88]
*Chlorocebus pygerythrus*	195	163	***0.84***	[102]
*Trachypithecus obscurus*	168	205^4^	**1.22**	[103]
*Hylobates lar*	210	190	***0.90***	[104]
*Pongo pygmaeus*	270	258	0.96	[105]
*Pan troglodytes*	230	229	1.00	[106]
*Gorilla gorilla*	265	255	0.96	[105]

A proportion of less than 1.0 indicates that – compared to Sacher & Staffeldt [32] – gestation length is now considered to be shorter in the respective species. A value over 1.0 indicates the opposite. Bold, underlined = current value 1.1 or more; bold italic = current value 0.9 or less. Note also that since 1974 gestation length has been determined for many more than these 15 species.

1 Species names standardized according to Groves [107].

2 Identical values in Sacher & Staffeldt [32] for two species.

3 The two values in Sacher & Staffeldt [32] were averaged.

4 Value for a closely related species of similar body mass, wild *Trachypithecus phayrei.*

What might have caused these large differences? Gestation length ***is*** a straightforward measure, lasting from conception to parturition [37]. The main problem seems to lie in how conceptions were determined [38] and with it the onset of gestation. Using the patterning of mating behavior alone can be misleading, because female primates often continue to mate when pregnant [39], [40], thus shortening the estimates for gestation length. Therefore, we selected only those more recent values (Table 1) for which conceptions were determined hormonally. Alternatively, if mating behavior was used, additional information had to be considered such as menstruation or patterns of sexual swellings [41]–[43]. These recent values are thus likely to be more accurate. Given improvements in measuring techniques such as non-invasive hormonal determination of conception and pregnancy [44], older databases may not provide the most accurate values. We will demonstrate below how such differences can greatly impact the outcome of comparative analyses.

### Interbirth Interval

In addition to ultimate effects of mortality on maturation [45], [46], infant death directly affects interbirth intervals. In most primates interbirth intervals are shorter after premature infant loss as a consequence of female reproductive physiology: suckling disrupts the release pattern of the gonadotrophin releasing hormone (GnRH) and of the luteinizing hormone (LH) so that the LH surge fails and ovulation does not occur [47]. This suppressing effect stops when an infant dies; females resume cycling and conceive, resulting in a shorter interbirth interval after infant loss. To demonstrate the uniformity of this effect for primates, we collated examples (Table 2) from all major primate radiations based on long-term studies of wild populations. We excluded annual breeders such as most strepsirrhines [48], [49] and species with postpartum conceptions such as callitrichines [50], because in these taxa the interbirth interval is largely independent of infant mortality [51], although exceptions seem to exist [52].

**Table 2:** The effect of premature infant loss on the subsequent interbirth interval (IBI) for example species from all major primate radiations in taxonomic order.

Species	IBI infant lost [mos]	IBI infant survived [mos]	IBI infant survived as proportion of IBI infant lost	Signif. level	Reference(s)
*Alouatta seniculus*	10.5 (?)	17.0 (135)	1.6	nt	[110]
*Ateles geoffroyi*	11.0 (3)	32.8 (21)	3.0	**	[111]
*Cebus capucinus*	12.6 (?)	27.0 (?)	2.1	***	[112], total N = 74
*Sapajus nigritus*	14.1 (17)	20.4 (34)	1.5	***	[113]
*Cercopithecus mitis*	17.6 (53)	30.7 (193)	1.7	***	[114]
*Macaca fuscata*	18.0 (6)	26.9 (42)	1.5	*	[115]
*Papio anubis*	6.9 (20)	13.4 (61)	1.9	***	[116]
*Papio cynocephalus*	15.0 (61)	20.8 (269)	1.4	***	[54]
*Theropithecus gelada*	17.6 (11)	28.4 (37)	1.6	***	[117]; Beehner & Bergman, pers. com.
*Procolobus tephrosceles*	11.9 (11)	27.5 (45)	2.3	nt	[118]
*Presbytis thomasi*	17.7 (38)	26.8 (28)	1.5	nt	[119]
*Semnopithecus schistaceus*	19.2 (16)	32.4 (45)	1.7	***	[120]
*Trachypithecus poliocephalus*	12.7 (4)	25.0 (23)	2.0	**	[121]
*Hylobates lar*	26.4 (9)	40.8 (22)	1.6	***	[122]
*Gorilla beringei*	24.3 (35)	48.1 (98)	2.0	**	[123]; test for smaller N in Stewart et al. [124]
*Pan troglodytes*	26.6 (?)	71.9 (?)	2.7	nt	Mahale [125], total N = 174

Data for the same, wild population; annual breeders excluded; sample sizes in parentheses, per species the largest sample size was selected; species names according to Groves [107] with the exception of *Sapajus* [108] and *Procolobus* [109]. Signif. level: difference was significant at * = *P≤*0.05, ** = *P*<0.01, or *** = *P*<0.001, nt = not tested.

Ideally all infant deaths prior to the mothers’ subsequent conception should be considered because the age at infant death is often related to the length of the subsequent interbirth interval [53]–[55]. However, interbirth intervals are also reported in relation to a specific age of the infant at death, or until the females’ resumption of cycling, cessation of nipple contact, or subsequent parturition. Here we used the data on infant mortality and interbirth intervals as they were available in the primary literature without attempting to standardize further.

Because it is easier to visualize, we calculated the proportion by which the mean interval after surviving infants is lengthened relative to the mean interval after early infant loss (Table 2). This proportion ranged from 1.4 to 3.0 times longer if the infant survived and was related to the length of the interbirth interval for surviving infants (Pearson’s *r* = 0.502, *P*<0.05) which itself is correlated with body mass [33]. The relationship improves if the potential outlier *Ateles* spp. is removed (Pearson’s *r* = 0.629, *P*<0.02) but the correlation coefficient is low, indicating that additional factors may be important. Besides the age at infant death other influencing factors could include ecological conditions such as food availability acting on the likelihood to resume mating and to re-conceive [56], [57].

Whenever the difference was tested within a population, the interbirth interval after infant loss was significantly shorter compared to the interbirth interval after surviving infants (Table 2). The reason why we illustrate this well-known effect here again is because – as incomprehensible as it may sound – most existing life history compilations do not specify infant survival for interbirth intervals. It appears that most values used combine intervals after surviving infants with intervals after infant loss. Such confounding factors are particularly problematic for cross-species analyses because they are not unbiased errors that simply add noise that makes it more difficult to detect patterns (i.e., increased Type II error). Instead, systematic but variable bias in a single direction (such as interbirth intervals that are always less than or equal to the “real” interbirth intervals) can also produce spurious results which incorrectly identify significant relationships (i.e., increased Type I error).

### Population-Specific Covariates

In addition to the issue of correctly (or at least consistently) measuring life history variables, factors such as nutrient availability are known to significantly influence life history values within a given species or population [58], [59]. If more food is available, growth and maturation processes are accelerated, and the interbirth interval is shortened. To illustrate the magnitude of this effect we contrasted data for three cercopithecoid primates (*Macaca fuscata*, *Papio cynocephalus*, *Semnopithecus* spp.; Table 3) for which the large sample sizes indicate reliable values (small random error [31]).

**Table 3:** Examples for the influence of nutrition on primate life history in three cercopithecoid primates.

Species	Variable	Dimension	Provis	Wild	Wild as proportion of Provis	Signif. level	Population and references
*Macaca fuscata*	Gestation	days	176.3 (9)	173.0 (17)	0.98	nt	Japan Monkey Center *versus* Kinkazan [126], [127]
	Age at first reproduction	months	72.0 (23)	110.4 (42)	1.53	nt	Koshima [128]
	birth rate	infants/females*years	0.62 (66)	0.20 (31)	0.32	***	Koshima [128]
	IBI	months	17.5 (770)	26.9 (42)	1.54	nt	Arashiyama *versus* Yakushima [115], [129]
*Papio cynocephalus*	Gestation	days	175 (>50)	177 (590)	1.01	nt	Southwest Foundation *versus* Amboseli [88], [130]
	Age at menarche	months	41.2 (14)	56.0 (56)	1.36	***	Amboseli [131]
	IBI	months	15.4 (?)	21.7 (?)	1.41	nt	Amboseli [132]
*Semnopithecus* spp.	Gestation	days	200.3 (31)	211.6 (7)	1.06	***	Jodhpur *versus* Ramnagar [133], [134]
	Age at last nipple contact	months	12.8 (11)	24.9 (23)	1.95	***	Jodhpur *versus* Ramnagar [134], [135]
	Age at first reproduction	months	42.5 (12)	80.4 (26)	1.89	***	Jodhpur *versus* Ramnagar [43], [134]
	IBI	months	17.2 (82)	32.4 (45)	1.88	***	Jodhpur *versus* Ramnagar [43], [120]

Provis = either provisioned by people or with access to crops, a dumpster or in captivity; wild = no access to human-made food. Sample sizes in parentheses. IBI = interbirth interval after surviving infant. Signif. level: difference was significant at *** = *P*<0.001, nt = not tested.

In these examples, gestation length, as a measure of the growth rate of the fetus, was very similar (although not identical) in provisioned and wild populations. The other life history variables varied much more and consistently in relation to food availability. In Japanese macaques (*Macaca fuscata)*, age at first reproduction, and interbirth interval (as well as birth rate) were at least 1.5 times faster in provisioned populations. In a comparison of provisioned and unprovisioned groups of baboons (*Papio cynocephalus*) from the same population, age at menarche was 36% older and the interbirth interval 41% longer in unprovisioned groups. These effects were even stronger (almost double) between provisioned and wild Hanuman langurs (*Semnopithecus* spp.). Thus, clearly identifying and accounting for nutritional conditions should help to eliminate confounding effects in comparative studies (see more details below).

### Effects of Life History Measurement Variation: Does it Make Any Difference?

The factors illustrated above are well established, but despite this fact, existing life history compilations only rarely provide information on provisioning or infant survival and if they do, the percentage of unspecified cases can be high. For example, of all 12,001 life history values listed for primates in the PanTHERIA database [17], 67% had no information on wild *versus* provisioned conditions and for 76% of the 462 interbirth interval entries, infant survival was not mentioned. Most compilations do not identify the specific conditions for a population. Consequently, to address issues such as the influence of nutrition, some studies used only data for captive animals [60], [61] or only for wild ones [62] but the effects of infant survival on interbirth intervals (Table 2) are rarely taken into consideration.

Does it matter that outdated data are being compared or data collected for populations living under very different nutritional conditions and mortality schedules? Will results be different if analyses were based on data that also allow controlling for confounding factors? In the following we argue that data quality does indeed matter, because with existing compilations even the most basic relationships may not be recovered and that by controlling for nutrition and survival different trends and new insights can be disclosed.

### The Test: Comparing Life History Patterns in Asian Colobines and Asian Macaques

To demonstrate potential effects of data quality and confounding factors on life history analyses we built upon our previous investigation of feeding adaptations and life history in Asian colobines and Asian macaques [63]. In this analysis we compared these two taxa because they likely evolved under similar ecological conditions [64], [65] and extant species have a similar body mass range [66], [67], which is advantageous for cross-taxa comparisons [68]. It allowed us to explicitly investigate the difference relative to body mass using datasets in which results did not qualitatively differ regardless of whether or not we used comparative phylogenetic methods (discussed below). We had compiled all data from the original literature (selection criteria below).

Because protein is essential for growth [69] and primates mainly consume it as leaves which are less seasonal in abundance than e.g., fruits [70] it has been predicted that folivorous primates should grow faster compared to frugivorous ones [61]. However, when comparing the folivorous colobines with the frugivorous macaques in our sample while controlling for body mass, we found a ***longer***, instead of a shorter gestation length in folivores while age at first reproduction and interbirth interval did not differ [63]. In Malagasy lemurs this trend was even more pronounced. In lemuriform folivores all life history variables examined were significantly ***longer***, not shorter, compared to frugivores [71].

Possible causes for slower or similar life histories in the folivorous taxa are discussed in the respective studies [63], [71] and are not our focus here. Instead we will try to reconstruct our results for Asian colobines and macaques based on data from the other existing life history compilations mentioned above. Proceeding from the assumption that all databases are of comparable accuracy, we expect to find longer gestation lengths in the colobines but similar age at first reproduction and similar length of the interbirth interval in both taxa. The general allometric relationship between life history variables and adult female body mass [72], [73] will be considered in the analyses and will also be used as an indicator of data quality because every dataset should produce a tight correlation with log10 body mass.

### Data Selection and Setup

In our previously published analysis we compiled data on gestation length, age at first reproduction, and interbirth interval from original publications [63]. We included only gestation lengths for which the time of conception had been determined by hormonal analyses or in combination with additional signs such as menstruation or swelling patterns. For age at first reproduction no further specifications were available and we simply included the largest dataset. For interbirth intervals we selected those after a surviving offspring and identified the five (of 26) for which infant fate was not specified.

If more than one value was available for a given species we selected the one based on the largest sample size and of best quality, deliberately ignoring intra-specific variation because it was not our focus. Some species could not be considered due to insufficient data quality or quantity (details in [63]). We distinguished gross nutritional differences such that each species could be represented by up to two values per life history variable, one for free-ranging and provisioned or captive (provisioned/captive) and one for free-ranging and unprovisioned (wild/unprovisioned) populations. We performed the comparison in two steps: In the first step, we averaged the two values per species (if available) because the nutritional regime was not identified or was only incompletely identified in the other datasets. In the second step, we considered nutrition as a categorical variable to illustrate how it may change the outcome of the test. In an additional third step we restricted the analysis of interbirth intervals to include only those measured after surviving infants. Due to missing metadata this was not possible for the other compilations, and one dataset [13] only contained the inverse variable “litters/year”, which is similar but not identical to interbirth intervals [74], and therefore was not used.

Data for the same three life history variables (i.e., gestation length, age at first reproduction, and interbirth interval) were extracted for as many Asian colobine and macaque species as available from the four other published life history compilations mentioned above (PanTHERIA downloaded: November 2010; Ernest downloaded: August 2011; Kappeler & Pereira; Rowe & Myers downloaded: October 2011). Across datasets, sample sizes per variable and for both taxa combined ranged from 15 to 26 species with our dataset falling within that range (20 to 22 species per variable). Within each of these datasets, identical values for a given species were considered only once to control for potentially repeated inclusions of the same data if more than one secondary source had been used for the compilation. For example, 97% of the entries for primates in PanTHERIA were not from primary but from several secondary sources, and Ernest used only secondary sources. If multiple, ***different*** values per species were given, they were averaged. Thus, for this comparison, each species was represented by one value per variable per database. In addition, for PanTHERIA we excluded 12 gestations between 9–49 days in length, which must be errors because in both Asian colobines and macaques the shortest mean values are above 160 days [63].

The dimensions were standardized to days (for gestation length), years (for age at first reproduction), or months (for interbirth interval) and then log10 transformed. Each dataset was tested separately with adult female body mass as log10 kg values [66], [67] as a covariate, and taxon (colobine *versus* macaque) as a categorical, independent variable using an analysis of covariance (ANCOVA) [75]. Additional analyses were performed on the Borries et al. dataset with nutrition (wild/unprovisioned *versus* provisioned/captive) and in case of interbirth intervals the analysis was rerun for only surviving infants.

All analyses were repeated using phylogenetic generalized least squares (PGLS [76]). This approach incorporates phylogenetic information into linear models to account for the statistical non-independence of residuals (e.g., sister species sharing a recent common ancestor are expected to be more similar to each other in all observations and in their deviation from general patterns than two species whose lineages diverged much longer ago). PGLS models allow for the estimation of Pagel’s lambda, a parameter which identifies the strength of a phylogenetic signal in a linear model [77], [78]. A value of zero indicates a negligible phylogenetic signal (PGLS results are identical to those of non-phylogenetic linear models) and a value of one indicating that the data are patterned according to expectations based on the phylogenetic relationships of the species under study. The phylogenetic branching sequence and branch lengths used in this analysis were based on the consensus phylogeny (version 3) of the 10 kTrees Project [79], modified following Meyer et al. [80] for the inclusion of *Presbytis hosei* and *Presbytis thomasi*. All analyses were conducted in R [81] and phylogenetic analyses used the ‘caper’ package [82].

### Results Using Different Data Compilations

**Figure 2:**
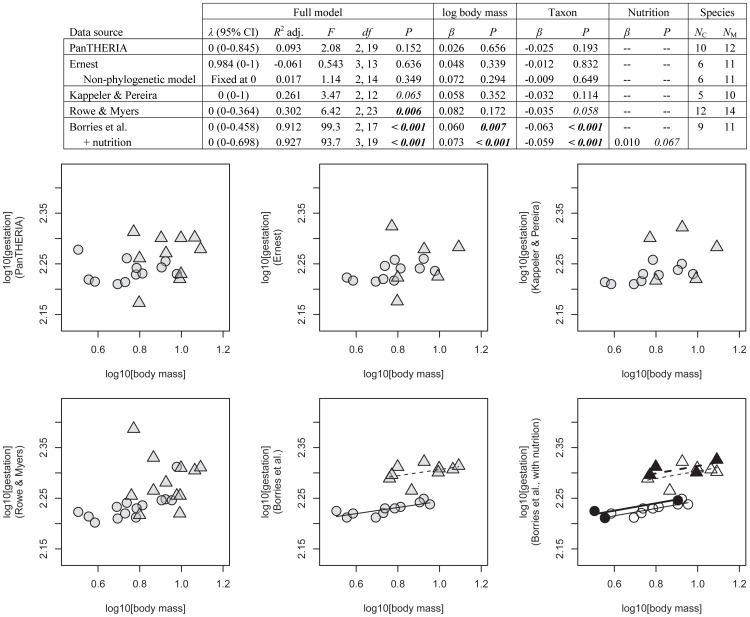
Gestation length [log10 days] in relation to adult female body mass [log10 kg] for different databases. Linear model results and plots for Asian colobines (triangles, dashed lines) and Asian macaques (circles, sold lines); *N*_C_ = number of Asian colobine species; *N*_M_ = number of Asian macaque species; – = N/A; bold italic = *P≤*0.05; italic = 0.05<*P*<0.10. Lines only drawn for models with a body mass effect that is significant at alpha = 0.05. Where nutrition is included as an independent variable, filled symbols and bold lines stand for wild/unprovisioned whereas open symbols and non-bold lines stand for provisioned/captive. Body mass data from Smith & Jungers [66] and Gordon [67].

Testing the effects of body mass and taxon on gestation length, PGLS results were identical to non-phylogenetic linear model results (lambda estimated as equal to zero) in all cases but one (Ernest for gestation length). In that case, neither the PGLS model nor the standard ANCOVA model were significant at alpha = 0.05 (*P = *0.636 and 0.349, respectively). When considering all five datasets, two of five models were significant and one revealed a trend (Fig. 2): with 91.2% the highest percentage of variance was accounted for by our dataset, while the second best model (Rowe & Myers) accounted for 30.2% of the variance. The third model, which accounted for 26.1% of the variance (Kappeler & Pereira) was a statistical trend (*P = *0.065). A significant body mass effect on gestation length was only documented in our dataset. Taxon had a significant effect in our dataset and there was a statistical trend in Rowe & Myers (*P = *0.058). If nutrition was also considered (our dataset only), the significance level for body mass improved while nutrition itself produced a statistical trend (*P = *0.067) but the variance accounted for by the model improved only very little (from 91.2% to 92.7%).

**Figure 3:**
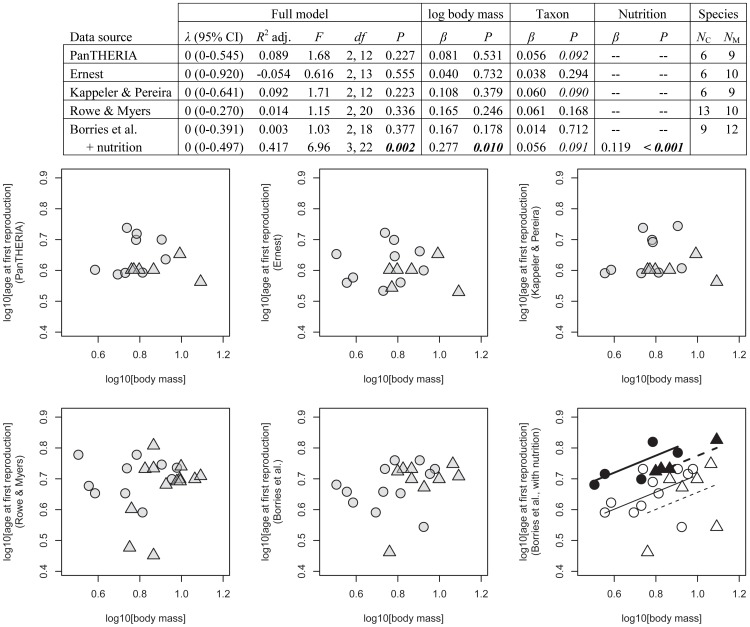
Age at first reproduction [log10 years] in relation to adult female body mass [log10 kg] for different databases. Symbols and abbreviations as in Fig. 2.

Testing the effects of body mass and taxon on age at first reproduction, PGLS results were identical to non-phylogenetic models, none of the five models were significant, and only a small percentage of the variance was accounted for (up to 9.2%, Fig. 3). There was no body mass effect on age at first reproduction in any of the datasets, while taxon produced a trend twice (PanTHERIA with *P = *0.092 and Kappeler & Pereira with *P = *0.090). In both cases age at first reproduction was older for macaques than for colobines. Once nutrition was also considered (our dataset only) the percentage of the variance accounted for improved from 0.3% to 41.7%, taxon produced a trend (*P = *0.091) with age at first reproduction older for macaques than for colobines, and body mass was significant as was the nutritional effect. In other words, only if the nutritional regime was considered did the result confirm the generally expected effect for body mass.

**Figure 4:**
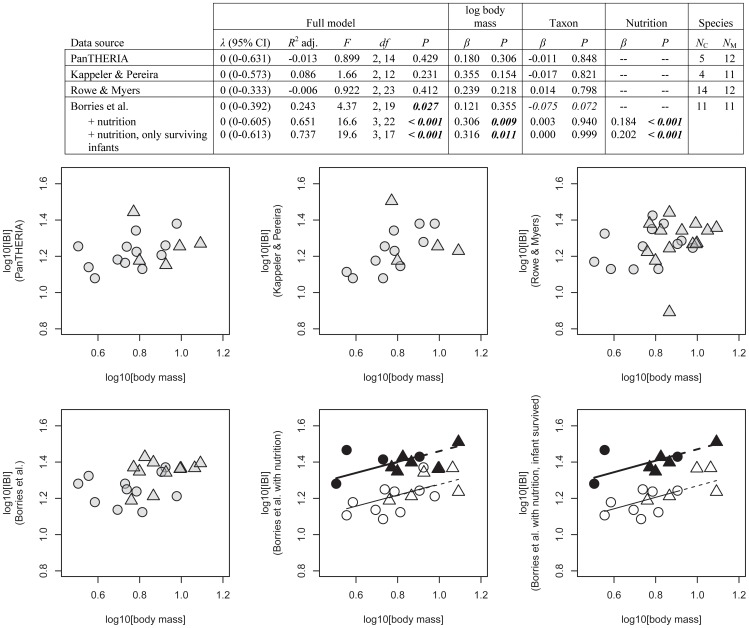
Interbirth interval, IBI [log10 months] in relation to adult female body mass [log10 kg] for different databases. Symbols and abbreviations as in Fig. 2. Note that dotted lines fall on top of the solid lines in the last two plots; i.e., the scaling relationships are very similar for Asian colobines and Asian macaques.

Testing the effects of body mass and taxon on interbirth intervals, PGLS results were again identical to non-phylogenetic linear model results. Only our model was significant, accounting for 24.3% of the variance (Fig. 4), the other models accounting for up to 8.6%. No significant body mass effect or taxon effect was found (besides a statistical trend for taxon in our dataset, *P = *0.072). Once nutrition was included (our dataset only) the variance accounted for improved from 24.3% to 65.1%. In the resulting model, body mass and nutritional effects both were significant while taxon was no longer a trend (Fig. 4). Using only interbirth intervals after surviving infants (i.e., reducing our dataset by 19.2% by excluding 5 of 26 values) did not alter the overall outcome, but the variance accounted for increased by 8.6 percentage points to 73.7%.

### When Sources of Variation are Ignored: Comparing Apples with Oranges

These results reveal striking differences between the databases considered even for detecting a body mass effect. In mammals, life history variables and adult female body mass are so strongly correlated [1] that body mass is considered a life history related variable [83]. The effect is particularly well documented for gestation length [35], [84], [85] but also holds for the other variables [86]. In the two taxa compared here the range in adult female body mass was small (∼3–12 kg) relative to all extant primates (<0.05 to >70.0 kg [66]), and to all extant mammals (<0.002 to >325 kg [87]) thus reducing the probability of recovering an effect in our sample. Still it was recovered for all three life history variables based on our dataset although twice only after additional inclusion of nutritional effects. In contrast, body mass ***never*** had the expected positive relationship with life history variables in any of the analyses from the other databases and in several cases the relationship with body mass was negative. These patterns were robust regardless of whether or not phylogenetic information was considered due to the generally negligible phylogenetic signal present in the residuals of the linear models.

The lack of a significant relationship between body mass and life history variables in most of the datasets considered here is exceedingly unlikely to reflect biological reality and, in our opinion, indicates severe problems with data quality. The body mass values used in this analysis cannot explain these phenomena as they were taken from the same sources [66], [67] and in each dataset the same species was assigned the same mean value.

Overall, the high explanatory power in the Borries et al. dataset seems to be in part due to our use of metadata related to nutrition as well as data selection based on standardized definitions of the respective variables. If nutrition was not controlled for, two of the three life history variables had no significant relationship with adult female body mass. This suggests a strong masking effect of nutrition. And indeed, nutrition was a significant factor for all three variables in our dataset. It was smallest for gestation length where it improved the variance accounted for by only one and a half percentage points (from 91.2% to 92.7%). For age at first reproduction the variance accounted for increased from 0.3% to 41.7% and for interbirth interval from 24.3% to 65.1%. The smaller impact on gestation length could be related to its shorter duration compared to interbirth interval and especially age at first reproduction. Alternatively or in addition, it could support the assumption that nutrition might have a weaker effect on prenatal compared to postnatal growth [34].

Furthermore, differences in variable definitions could be one of the key factors leading to discrepancies between different life history analyses. Of the three variables considered here, gestation length is the best example. Its definition is not questioned as it is the period from conception until birth. The main problem with gestation length derives from how it is measured. Especially in anthropoid primates, questionable data can result depending on how conceptions were determined [38]. Ideally, they are determined hormonally. In addition, the value reported for gestation length can vary with infant viability. For example in wild baboons (*Papio cynocephalus* [41]) gestation length ranged from 154 to 209 days, thus spanning 56 days. This range shrank to 20 days (164 to 183 days) if neonatal deaths and stillbirths were excluded. The effect was later confirmed for the population with a very large sample size (*N* = 590 [88]). Thus, while often difficult to apply in practice, using only standardized life history variables in comparative analyses should be an important goal for the future.

Lastly, as illustrated in Table 2, the interbirth interval is significantly influenced by infant mortality especially in non-annually breeding species characterized by a long lactational amenorrhea relative to gestation length [89]. Consequently, in case all interbirth intervals of a population are considered, the mean value will also reflect the local pattern of infant mortality. However, this is counterproductive because the variable should only capture the time it takes to raise an offspring until the next one is born.

Without quality filters and clear variable definitions we risk comparing apples with oranges, or perhaps Red Delicious with Granny Smith – two fruits which are clearly both apples, and yet also quite distinct from each other. Similarly, small but clear differences in the definition of the life history variables compared are likely the reason why the agreement between the results from the different databases is so poor.

## Conclusions

### We Need to Start Over!

Thus, the answer to our initial question is “no”: the results for Asian colobines and macaques from our analysis [63] could not be reproduced based on the other four datasets. Had we used any of the other existing datasets and not compiled our own, we would still be unaware that gestation length in Asian colobines is significantly longer than in Asian macaques. Furthermore, we would not be confident that interbirth intervals did not differ in the two taxa. These results contrast with the widely held belief that compared to Asian macaques, the more folivorous Asian colobines have a shorter interbirth interval in line with growth measures for captive primates [61], although there is some weak support (a statistical trend) for the idea that colobines have a younger age at first reproduction than macaques. Overall, our present comparisons strongly indicate that there can be marked discrepancies in the results depending on which database was used. Based on standardized variables and by controlling for a few key factors, new insights were gained about life history and dietary adaptations [63]. Together with similar trends discovered for strepsirrhines [71] it is now likely that folivory does not facilitate growth as much as had been suggested previously.

Perhaps even the general role assumed for protein intake with respect to primate growth needs to be reconsidered. A recent study on juvenile rhesus monkeys emphasized the importance of caloric over protein intake [90]: Females kept on a low caloric-high protein (70%) diet reached menarche significantly later than those on a high caloric-low protein (19%) diet. The same effect is also described for humans in connection with juvenile obesity [91].

More generally, at present we do not know if the results of other past life history analyses would hold if based on data that were cleaned-up in a way described here. Clearly this will also depend on the taxonomic level of analysis. Errors of the magnitude identified above might not matter (or matter less) when primate life histories are contrasted with those for rodents because the very large differences should make for strong signals. However, the taxonomic level at which data quality issues no longer have a noticeable effect on results can only be found by trial and error, by re-running analyses at various taxonomic levels and data accuracies. It could be that in the future key aspects at least of primate life history might have to be viewed very differently, with potentially far reaching consequences [25], [92]. It could also be that new directions recently taken in primate life history analysis will have to be revisited such as interpreting differences in the scaling with body mass between gestation length, weaning age and development time (i.e., gestation plus lactation) based on metabolic effects [23].

### A New Life History Database

In the past, few data for primate life history were available and compilations seem to have been built in a catch-as-catch-can fashion. Researchers were also mostly unaware of the magnitude of the influence that conditions such as nutrient availability could have. This is still reflected in the content of most of the existing compilations, which often build on one another such as the data from Ernest analyzed here which – with respect to primates – rely heavily on the Kappeler & Pereira dataset. In the future, researchers might perhaps want to pay more attention to the quality of existing data, whether they meet the selection criteria required for the analysis planned. For example, if infant survival is not specified for interbirth intervals these data can likely not answer most of our questions. We can no longer unconditionally use what is published.

That said, there is a tremendous amount of primate life history data currently available, much or most of it usable in comparative analyses given adequate associated metadata. Therefore we propose building a new, publicly available database. Such a database would specify variable definitions and methods of assessment; it would provide mean values with descriptive statistics, and include metadata on nutritional conditions, infant viability and the like. Many details still have to be worked out before we can reach a standardized level and certain criteria need to be developed with time and practice. For example, when considering nutritional regimes it could be that in the end we can only distinguish between two very basic regimes such as those we did in our current analysis: namely whether or not human-made food was ever consumed.

Certainly there is also individual variation and there is variation between sites and between species, and such a database that recorded information at the population level would identify and preserve the differences. In addition it is also reasonable to identify a “best value” (central tendency) for every population (and condition) for use in comparative analyses. We need to locate and compile those values, have them proof-read and approved by the people who collected the data, and keep the compilation updated so that future research can be based on the best data currently available. Ideally, in the – hopefully not too far – future new data will automatically be included at the time of publication. Other disciplines already have comparable structures in place such as databases like GenBank® (http://www.ncbi.nlm.nih.gov/genbank) and standards for database variables like Darwin Core (http://www.gbif.org). Such a life history database is likely to save us all a lot of time in the future and will allow for new and true discoveries.

As a final note: We used Asian primate taxa as example because it is the group we are most familiar with. Similar trends and issues as those raised here likely also hold for the other primate taxa and other animal orders and their databases. Try it out. The results might surprise you.
